# Parallel Reaction Monitoring Mass Spectrometry for Rapid and Accurate Identification of β-Lactamases Produced by *Enterobacteriaceae*

**DOI:** 10.3389/fmicb.2022.784628

**Published:** 2022-06-20

**Authors:** Yun Lu, Xinxin Hu, Jing Pang, Xiukun Wang, Guoqing Li, Congran Li, Xinyi Yang, Xuefu You

**Affiliations:** Beijing Key Laboratory of Antimicrobial Agents, Institute of Medicinal Biotechnology, Chinese Academy of Medical Sciences and Peking Union Medical College, Beijing, China

**Keywords:** *Enterobacteriaceae*, β-lactamases, specific peptides, detection, PRM

## Abstract

The increasing spread of drug-resistant bacterial strains presents great challenges to clinical antibacterial treatment and public health, particularly with regard to β-lactamase-producing *Enterobacteriaceae*. A rapid and accurate detection method that can expedite precise clinical diagnostics and rational administration of antibiotics is urgently needed. Targeted proteomics, a technique involving selected reaction monitoring or multiple reaction monitoring, has been developed for detecting specific peptides. In the present study, a rapid single-colony-processing procedure combined with an improved parallel reaction monitoring (PRM) workflow based on HRAM Orbitrap MS was developed to detect carbapenemases (*Klebsiella pneumoniae* carbapenemase, KPC; imipenemase, IMP; Verona integron-encoded metallo-β-lactamase, VIM; New Delhi metallo-β-lactamase, NDM; and oxacillinase, OXA), extended spectrum β-lactamases (TEM and CTX-M), and AmpC (CMY-2) produced by *Enterobacteriaceae*. Specific peptides were selected and validated, and their coefficients of variation and stability were evaluated. In total, 188 *Enterobacteriaceae* strains were screened using the workflow. Fourteen out of total 19 peptides have 100% specificity; three peptides have specificity >95% and two peptides have specificity ranged from 74∼85%. On the sensitivity, only nine peptides have 95∼100% sensitivity. The other 10 peptides have sensitivity ranged from 27∼94%. Thus, a screening method based on peptide groups was developed for the first time. Taken together, this study described a rapid extraction and detection workflow for widespread β-lactamases, including KPC, IMP, VIM, NDM, OXA, CMY, CTX-M, and TEM, using single colonies of *Enterobacteriaceae* strains. PRM-targeted proteomics was proven to be a promising approach for the detection of drug-resistant enzymes.

## Introduction

With the extensive clinical use of carbapenems and β-lactam antibacterial drugs, the incidences of antibiotic resistance have increased. The increasing population of multi-drug resistant (MDR) and extensively drug-resistant strains accompanied with the rapid spread of antibiotic-resistance genes have posed great challenges to clinical anti-bacterial treatment and public health. In the list of bacteria for which new antibiotics are urgently needed released by the World Health Organization in 2017, carbapenem-resistant *Enterobacteriaceae* (CRE) and extended-spectrum β-lactamase (ESBL)-producing *Enterobacteriaceae*, were included in the Critical group (Priority 1) (WHO, 2017). β-lactamases are a group of bacterial enzymes that can inactivate β-lactam antibiotics, resulting in the loss of antibacterial activity (Eliopoulos and Karen, 2001). β-lactamases are currently divided into four classes: A, B, C, and D according to the Ambler classification, based on their primary structure (Bush and Jacoby, 2010). Carbapenems are generally regarded as the last treatment choice for serious bacterial infections. Carbapenemases are β-lactamases with versatile hydrolytic capacities: the A and D class carbapenemases are serine-type hydrolases, such as *Klebsiella pneumoniae* carbapenemase (KPC), and oxacillinase (OXA). The B class carbapenemases are metallo-hydrolases, such as New Delhi metallo-β-lactamase (NDM), imipenemase (IMP), and Verona integron-encoded metallo-β-lactamase (VIM). ESBLs belong to the class A and D β-lactamases. ESBLs such as the TEM (ampicillin resistance) and CTX-M (cefotaxime resistance) groups belong to class A β-lactamases (Pitout et al., 2005). AmpC β-lactamases such as CMY-2 belong to class C of carbapenemases (Philippon et al., 2002). *Enterobacteriaceae*, including *Escherichia coli*, *K. pneumoniae*, and *Enterobacter cloacae*, which carry several types of β-lactamases, represent a great challenge to the clinical diagnostics and treatment of various infections (Duin, 2017).

Early diagnosis and effective drug treatment are key strategies to deal with the antibiotic-resistance problem (Rodríguez-Baño et al., 2018). Thus, rapid and accurate detection methods are urgently needed in clinical practice. A short testing time and accurate diagnosis will assist in providing an appropriate antibiotic treatment in time. For the last few decades, traditional approaches for the detection of β-lactamases, such as standard disk-diffusion procedure, broth microdilution, and agar dilution, have been used, which are time-consuming and can only determine the drug-resistance property but not the β-lactamase type. Synergy testing is accurate and allow to identify the carbapenemase classes, which is the info needed for therapeutic choices. Polymerase chain reaction (PCR) are very sensitive and fast and can be used also from primary samples (Dallenne et al., 2010). Whole genome sequencing, which can accurately identify the genotypes of β-lactamases, is still not practical for usual clinical application because of the high price. However, since drug-resistant enzymes are the products of regulated expression, the detection of a gene may not be correlated with the successful expression of the β-lactamases. Carba NP test based on the detection of enzyme activity (Vasoo et al., 2013) are rapid but not specific for a single type of carbapenemase. The mCIM and eCIM, phenotypic detection methods based on carbapenem inactivation methods, can detect carbapenemases in *Enterobacteriaceae* and *Pseudomonas aeruginosa* and differentiate metallo-beta-lactamases from serine carbapenemases in *Enterobacteriaceae* (Tsai et al., 2020).

The direct detection and quantification of β-lactamases have become easier with the development of protein detection and quantitation techniques in recent years. Lateral flow immunoassay methods based on antigen-antibody reaction (Boutal et al., 2017) are limited to the types of antibodies, but actually they cover the vast majority of carbapenemases found in clinical microbiology routine, beyond being extremely fast. For the past few decades, liquid chromatography with tandem mass spectrometry (LC-MS/MS) has been widely used in various fields of protein analysis, biochemical analysis, natural product analysis, and drug and food analysis among other areas (Suh et al., 2017). LC-MS/MS has gradually become one of the most popular analytical tools for protein detection. Shotgun proteomics has been used to identify wild-type and resistant strains of the pathogen *Acinetobacter baumannii* (Chang et al., 2013). Additionally, a capillary electrophoresis-electrospray ionization-tandem mass spectrometry bottom-up proteomics workflow has been established for the identification of OXA-48 and KPC (Fleurbaaij et al., 2014). MALDI-TOF MS has been used to identify for carbepanemase detection with different analytical approaches (Hleba et al., 2021). Additionally, the bottom-up proteomics approach has also been applied to identify CTX-M ESBLs (Fleurbaaij et al., 2017). Recently, targeted LC-MS/MS based on selected reaction monitoring (SRM) and multiple reaction monitoring (MRM) using triple quadrupole mass analyzer, and parallel reaction monitoring (PRM) techniques using on high resolution/accurate mass has been used in β-lactamase testing. Specific peptides of *A. baumannii* identified *via* LC-MS/MS profiling have been used to classify clinical isolates (Honghui et al., 2016). Targeted high-resolution MS assays have been developed for the detection of KPC, OXA-48, NDM, and VIM enzymes (Wang et al., 2017, 2019; Foudraine et al., 2019; Strich et al., 2019). However, there are still many β-lactamases that have not been detected by targeted proteomics. Traditional SRM-MS on a triple quadrupole mass spectrometer is limited in the complex sample analysis due to the mass filtering and low resolution quadrupole, and more method development time is needed to define transitions (precursor/product ion pairs). In our study, a system comprising a rapid sample-processing procedure combined with improved PRM using HRAM (High resolution accurate mass) Orbitrap MS was developed to detect carbapenemases (KPC, IMP, VIM, NDM, and OXA), ESBLs (TEM and CTX-M), and AmpC (CMY-2) using a single colony of *Enterobacteriaceae* strains.

## Materials and Methods

### Strains and Culture Conditions

A total of identified and subcultured 192 *Enterobacteriaceae* strains were used in this study (Supplementary Table 3), including *K. pneumoniae* American Type Culture Collection (ATCC) BAA-2146 and 191 clinically isolated strains. The selected strains comprised 73 *E. coli*, 83 *K. pneumoniae*, 25 *E. cloacae* strains, five *Klebsiella oxytoca*, and six *Citrobacter freundii.* Four of these isolates were used for the test development process, and 188 were used for the test validation phase. All the strains were cultured in Luria-Bertani agar plates at 37°C overnight. All the strains were stored in the Collection Center of Pathogen Microorganism of Chinese Academy of Medical Sciences in China.

### Peptide Preparation

Single colonies (diameter >2 mm) were picked using a micropipette tip or a 10 μL loop, and resuspended in 200 μL of 50 mM ammonium bicarbonate (MS grade; Merck, Germany), sonicated for 1 min (3 s of sonication, 6 s of rest), centrifuged at 12,000 × *g* for 2 min and heated at 95°C for 5 min, after which the buffer was removed using 10K Nanosep centrifugal device with Omega membrane (Pall Corporation, Port Washington, NY, United States). Ammonium bicarbonate buffer (50 mM) was added along with sequencing grade trypsin (Promega Corporation, Madison, WI, United States), and the solution was microwaved in a water bath followed by heat treatment at 55°C. The peptide concentration was measured using the Pierce™ Quantitative Colorimetric Peptide Assay kit (Thermo Fisher Scientific, Waltham, MA, United States).

### Nano LC-MS/MS

Data-dependent analysis was performed on the Thermo Scientific Orbitrap Fusion Lumos platform coupled with an EASY-nLC 1200 system (Thermo Fisher Scientific, San Jose, CA, United States) to build a spectral library. The digests were separated by the trap column [ReproSil-Pur 120 C18-AQ (3 μm, Dr. Maisch GmbH, Ammerbuch, Germany); 20 × 0.05 mm] followed by a C18 column [ReproSil-Pur 120 C18 (1.9 μm, Dr. Maisch GmbH, Ammerbuch, Germany); 120 × 0.15 mm] at a flow rate of 600 μL/min. The solvent buffer A comprised water with 0.1% formic acid, and solvent B comprised 80% acetonitrile with 0.1% formic acid. After sample loading, the gradient was initiated with 11% of buffer B, and then increased from 11 to 13% of buffer B for 2 min. The gradient increased up to 32% of buffer B in 16 min, and then to 42% in 7 min. Finally, the gradient was increased to 95% of buffer B in 1 min and was maintained for 4 min. The MS parameters were as follows: MS1 (Orbitrap analysis; mass range, approximately 350–1,550 m/z; resolution, 120,000; AGC target, 5 × 10^5^; RF lens, 50%; maximum injection time, 50 ms), MS2 [high-energy collisional dissociation (HCD) collision energy, 32%; maximum injection time, 22 ms; AGC, 5 × 10^4^; isolation window, 1.6 Da; Orbitrap solution, 15,000]. For database analyses, these raw data were searched against a combined bacterial antimicrobial resistance database downloaded from the National Center for Biotechnology Information (NCBI; National Institutes of Health, Bethesda, MD, United States) using the Thermo Scientific™ Proteome Discoverer™ version 2.2 (PD2.2) software. Trypsin was specified for protein digestion with two missed cleavages allowed for each peptide. The search parameters were set as previously described (Espadas et al., 2017).

### Peptide Selection

The protein sequences of KPC, VIM, IMP, NDM, OXA, TEM, CMY, and CTX-M were downloaded from the NCBI database^1^ to build an amino acid sequence library. Tryptic peptides were searched and aligned using the protein basic local alignment search tool (BLAST^2^) to ensure uniqueness. The peptides with missed-cleaved, two neighboring basic amino acids at either cleavage site (KK, RR, KR, and RK), and W and M residues were excluded.

### Targeted Proteomics and Data Analysis

Targeted proteomics was performed on the Thermo Scientific Orbitrap Fusion Lumos platform coupled with an EASY-nLC 1200 platform. QQDLVDYSPVSEK and VDAGDEQLER served as internal reference. The columns used and elution gradient were the same as mentioned above. PRM parameters: MS1 spectrum (Orbitrap analysis; resolution, 60,000; mass range, approximately 350–2000 m/z; RF lens, 30%; AGC target, 2.0 × 10^5^; maximum injection time, 50 ms) and MS2 analysis (HCD; collision energy, 30%; AGC, 5.0 × 10^4^; maximum injection time, 54 ms for specific peptides or 22 ms for synthetic isotope labeled (SIL) peptides; Orbitrap resolution, 30,000 for specific peptides or 7,500 for SIL; isolation window, 1.4 Da). Data on the peptides [retention time (RT), m/z, and charge] were imported into the MS method. For data analysis, the acquired data were analyzed using Skyline 20.1.0.155 (MacCoss Lab Software, University of Washington, Seattle, WA, United States) (MacLean et al., 2010). The amino acid sequences of the drug-resistant enzymes downloaded from the NCBI were imported as the background library. Data-dependent acquisition (DDA) raw data were imported to build the spectra database. After the amino acid sequences of specific and SIL were inserted, the PRM data were imported and for each targeted peptide, the ratio between the peak area of the endogenous peptide and that of the SIL was calculated, and the relative concentration of targeted peptides was calculated based on the SIL with fixed quantity. Whether the CRE/ESBL enzymes were defined as positive or negative depended on the peptide when the following criteria were met: an RT similar to that of the SIL, library dot product (dotp) > 0.8 and ratio dot products (rdotp) > 0.95. Coefficients of variation (CV) were calculated, and stability of the peptides was evaluated by performing three freeze-thaw cycles of the peptides and storage of the peptides for 0, 1, 3, and 4 days in the sample holder (10°C).

### Detection of Drug-Resistant Genotypes *via* Polymerase Chain Reaction

Multi-drug resistant genes were analyzed *via* PCR (Dallenne et al., 2010) using the GoTaq Green Master Mix (Promega). The primers and parameters used are listed in Supplementary Table 1.

## Results

### Selection of Unique Peptides

Amino acid sequences of the enzymes were downloaded from the NCBI,^3^ and potential peptides were evaluated using PeptideCutter.^4^ In view of the varying responses of peptides analyzed *via* MS, four strains (Table 1) were used to evaluate the ionization capabilities of peptides *via* DDA, and the specificity of the peptides was assessed by performing BLASTp searches. Unique peptides with high signal stability, appropriate RT, and relatively stable amino acid residues were chosen as peptides markers for KPC, IMP, VIM, NDM, OXA, CMY, CTX-M, and TEM. Val (13C_5_, 15N), Gly (15N), and Ala (13C_3_, 15N) were used to label the peptides (Table 2). Other candidate peptides detected are listed in Supplementary Table 2. The data are deposited in the PRIDE repository, accession number PXD028791.

**TABLE 1:** β-lactamase information of isolates used in method development.

Name	KPC	NDM	VIM	IMP	OXA	TEM	CMY	CTX-M
*Klebsiella pneumoniae* ATCC BAA-2146 (Kwon et al., 2016)	–	+	–	–	+	+	+	+
*Klebsiella pneumoniae* 1705^a^	+	+	–	–	+	+	–	+
*Klebsiella pneumoniae* 17-R66^a^	–	–	–	+	–	+	–	+
*Klebsiella pneumoniae* 17-R42^a^	–	–	+	+	–	+	–	–

*^a^Measured by PCR and DNA sequencing.*

*KPC, Klebsiella pneumoniae carbapenemase; IMP, imipenemase; VIM, Verona integron-encoded metallo-β-lactamase; NDM, New Delhi metallo-β-lactamase; OXA, oxacillinase; CTX-M, β-lactamase against cefotaxime.*

**TABLE 2:** Selected target peptides for the rapid detection of multi-drug resistance enzymes.

Peptide	Enzyme	Genotype^a^	Labeled site	Charge	m/z (unlabeled)	RT (min)
AAVPADWAVGDK	KPC	1∼95	GLY(15N)	2	600.3064	15.4
SQQQAGLLDTPIR	KPC	1∼95 except for 13, 45, 59	GLY(15N)	2	713.8861	16.7
LVVPSHSEVGDASLLK	IMP-1	1, 5, 7, 10, 28, 30, 34, 40, 42, 43, 52, 55, 60, 61, 66, 70, 73, 76, 77, 79, 81, 85, 88	GLY(15N)	2	825.9567	16.1
VQATNSFSGVNYWLVK	IMP-1	1, 3, 6, 10, 25, 30, 34, 40, 42, 52, 55, 60, 61, 66, 70, 76∼80, 88	GLY(15N)	2	906.9676	22.9
NSFGGVNYWLVK	IMP-4	4, 26, 38, 59, 89	GLY(15N)	2	692.3564	22.6
LDEGVYVHTSFEEVNGWGVVPK	IMP	1, 3, 4, 5, 6, 7, 15, 25, 28, 29, 34, 38, 51, 52, 60, 61, 62, 59, 70, 79, 81, 82, 85	GLY(15N)	3	821.0727	22.2
LAEAEGNEIPTHSLEGLSSSGDAVR	VIM	1, 4, 5, etc.	GLY(15N)	3	847.0805	16.33
DGDELLLIDTAWGAK	VIM	All except for 7, 13, 47, 61, 69	GLY(15N)	2	808.9120	26.71
AFGAAFPK	NDM	1∼31	GLY(15N)	2	404.7212	14.6
NNGLTEAWLESSLK	OXA-1 family	1, 4, 31, 47, 224, 320, 392, 534, 675(oxa-1 family)	GLY(15N)	2	781.3965	22.3
IINHNLPVK	OXA-1 family	1, 4, 31, 47, 224, 320, 392, 534, 675(oxa-1 family)	VAL (13C5, 15N)	3	349.8818	8.8
ADIANNHPVTQQTLFELGSVSK	CMY-2 family	2, 4, 5, 6, 7, etc.	GLY(15N)	2	1185.108	19.5
TLQQGIALAQSR	CMY-2 family	2, 4, 5, 6, 7, etc.	GLY(15N)	3	429.2454	14.2
QLTLGHALGETQR	CTX-M-9 group	9, 13, 14, 17, 21, 19, 24, 65, 81, etc.	GLY(15N)	3	475.2599	13.3
TEPTLNTAIPGDPR	CTX-M	All ctx-m genotypes except for 4, 6, 7, 19, 23, 35, 42, 52, 54, 58, 62, 74, 87, 93, 99, 117, 126, 144, 147, 151, 155, 157, 168, 204, 219, 212, 221	GLY(15N)	2	741.3834	15.1
LIAQLGGPGGVTAFAR	CTX-M-9 group	9, 13, 14, 16, 21, 17, 19, 81, etc.	GLY(15N)	2	764.4357	20.8
APLILVTYFTQPQPK	CTX-M-1 group	1, 3, 10, 12, 15, etc.	Ala (13C3,15N)	2	858.4902	26.8
SDLVNYNPIAEK	CTX-M-1 group	1, 2, 5, 12, 15, etc.	Ala (13C3,15N)	2	681.8486	16.1
VGYIELDLNSGK	TEM	All except for 60, 139, 178, 210	GLY(15N)	2	654.3457	19.56

*^a^All the genotype-matching results are based on data obtained from the NCBI ANTIMICROBIAL RESISTANCE GENE database. With an increase in data, the results will change. m/z, mass to charge ratio; RT, retention time.*

### Parallel Reaction Monitoring Assay Development

**FIGURE 1:**
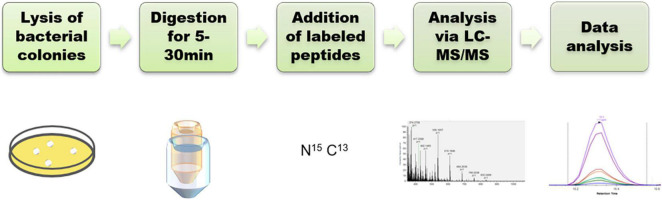
Workflow for the detection of β-lactamases.

**FIGURE 2:**
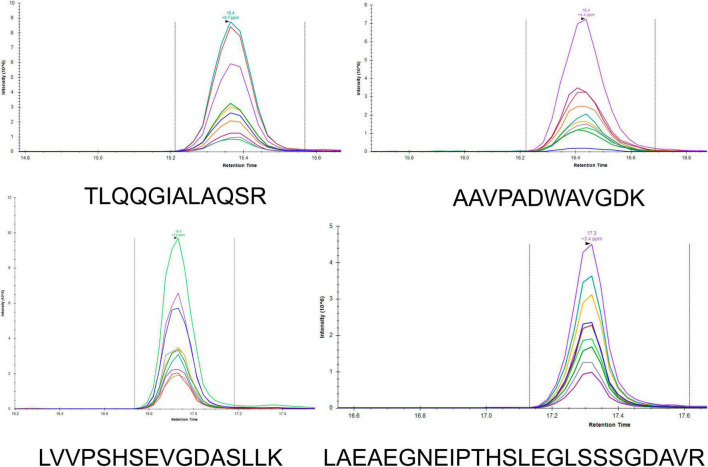
LC-MS/MS chromatograms of the peptides (TLQQGIALAQSR, AAVPADWAVGDK, LVVPSHSEVGDASLLK, and LAEAEGNEIPTHSLEGLSSSGDAVR) in Skyline. Different colors represent different fragment ions.

To develop a rapid method for peptide detection, a 30-min Nano LC-MS/MS method was developed, with parameters for peptide markers as shown in Table 2. Different Orbitrap resolutions for specific and SIL were used to improve identification speed, quantity and quality (Stopfer et al., 2021). Figure 1 shows the workflow of the rapid detection method for β-lactamases. The raw data were analyzed using Skyline, and the library dotp, rdotp, and R ratio values were exported. According to the library dotp, rdotp, and R ratio values obtained from the strains, the following rules were set: library dotp > 0.8, rdotp > 0.95, and RT similar to that of SIL (Figure 2). A wash procedure for 30 min was performed after each sample to avoid false-positive results caused by the carryover effect.

### Optimization of Rapid Digestion Conditions

To obtain high-quality spectra while reducing the digestion time, protein solutions with trypsin were microwaved for 5, 10, and 15 min, as well as heated in a water bath at 55°C for 15, 30, and 45 min after being microwaved for 2 min separately. Peptides were collected and analyzed *via* Nano LC-MS/MS. The total processing time was <1 h. As shown in Supplementary Figure 1, the peptides could be detected for all digestion conditions even after being microwaved for 5 min. For most peptides, an increase in digestion time led to an increase in abundance; however, the digestion conditions had no effect on the peptides LAEAEGNEIPTHSLEGLSSSGDAVR, VQATNSFSGVNYWLVK, IINHNLPVK, and NSFGGVNYW LVK. To ensure that all peptides were identified under optimal conditions, microwave treatment for 2 min combined with heat treatment using a 55°C water bath for 30 min was performed in subsequent experiments.

### Reproducibility and Stability Tests

The reproducibility of applications of the peptide markers was evaluated using four strains that were positive for KPC, IMP, VIM, NDM, OXA, CMY, CTX-M, and TEM based on the CV of six replicates in 1 day and in 3 different days (Table 3). The CVs of endogenous contents were determined using SIL (Table 2). The CVs of 16 peptides were <30%. While the CV of IINHNLPVK wasn’t acquired as signal miss in some of the samples. No carry-over effects were observed for any of the selected peptides.

**TABLE 3:** Intensity ratios and coefficients of variation of peptides.

Enzyme	Peptide	CV (%) in 1 day	CV (%) in 3 different day
KPC	AAVPADWAVGDK	23	25
KPC	SQQQAGLLDTPIR	12	12
IMP-1	LVVPSHSEVGDASLLK	8	11
IMP-1	VQATNSFSGVNYWLVK	10	12
IMP-4	NSFGGVNYWLVK	9	20
IMP	LDEGVYVHTSFEEVNGWGVVPK	9/14	9/20
VIM	LAEAEGNEIPTHSLEGLSSSGDAVR	5	8
VIM	DGDELLLIDTAWGAK	1	2
NDM	AFGAAFPK	14/27	23/10
OXA-1 family	NNGLTEAWLESSLK	6/21	5/17
OXA-1 family	IINHNLPVK	*	*
CMY-2 family	ADIANNHPVTQQTLFELGSVSK	15	17
CMY-2 family	TLQQGIALAQSR	13	14
CTX-M-9 group	QLTLGHALGETQR	11	15
CTX-M	TEPTLNTAIPGDPR	6/6/17	9/13/9
CTX-M-9 group	LIAQLGGPGGVTAFAR	15/16	14/24
CTX-M-1 group	APLILVTYFTQPQPK	37/72	33/57
CTX-M-1 group	SDLVNYNPIAEK	36/42	32/36
TEM	VGYIELDLNSGK	3/6/13	11/5/4

CV, coefficient of variation; *IINHNLPVK was not detected in both samples.

Stability is an important property that should be evaluated in method development. The SIL were added to the peptide solutions of *E. coli* DH5α that did not contain β-lactamases. The peptide stability after three freeze-thaw cycles and stability in the sample holder were measured separately. Six replicates were performed for all experiments. As shown in Supplementary Figure 2, the contents of DGDELLLIDTAWGAK and VGYIELDLNSGK decreased significantly after three freeze-thaw cycles (<80%), whereas the contents of LIAQLGGPGGVTAFAR and APLILVTYFTQPQPK decreased slightly (approximately 80–90%). These results suggest that repeated freezing and thawing of these four peptides should be avoided during use. Regarding stability in the sample holder for 0, 1, 3, and 4 days, the content of DGDELLLIDTAWGAK decreased significantly (70%) in 1 day, and down to 6% on the fourth day (Supplementary Figure 3). The content of VGYIELDLNSGK decreased significantly (64%) on the third day, whereas the content of APLILVTYFTQPQPK reduced to approximately 82% on the fourth day. The results suggest that these peptides should be held for short durations in the sample holder.

### Method Validation

After the preliminary evaluation of the rapid detection method, blinded testing of 188 clinical strains was conducted. Strains of *E. coli*, *K. pneumoniae, E. cloacae* strains, *K. oxytoca*, and *C. freundii* were included in the assays. As shown in Table 4, all the β-lactamases tested were detected both *via* LC-MS/MS and PCR. Most of the peptide markers for the β-lactamases showed 100% specificity except for LDEGVYVHTSFEEVNGWGVVPK(IMP, 85%), TEPTLNTAIPGDPR(CTX-M-1 group and 9 group, 97%), APLIL VTYFTQPQPK(CTX-M-1 group, 99%), SDLVNYNPIAEK (CTX-M-1 group, 95%), and VGYIELDLNSGK(TEM, 74%). Peptide markers for KPC(AAVPADWAVGDK), IMP-1, VIM-1, and NDM showed 100% sensitivity. However, the positive sensitivities for SQQQAGL LDTPIR(KPC), NSFGGVNYWLVK(IMP-4), LDEGVYVHTS FEEVNGWGVVPK(IMP), NNGLTEAWLESSLK(OXA-1), II NHNLPVK(OXA-1), ADIANNHPVTQQTLFELGSVSK(CMY-2), TLQQGIALAQSR(CMY-2), QLTLGHALGETQR(CTX-M-9 group), LIAQLGGPGGVTAFAR(CTX-M-9 group), TEPTLN TAIPGDPR(CTX-M-9 group, CTX-M-1 group partial), APLILVTYFTQPQPK(CTX-M-1 group), SDLVNYNPIAEK (CTX-M-1 group partial), and VGYIELDLNSGK(TEM) were 98% (49/50), 58% (14/24), 92% (24/26), 78% (14/18), 78% (14/18), 27% (6/22), 82% (18/22), 39% (27/70), 81% (57/70), 98% (116/118), 98% (57/58), 76% (44/58), and 74% (67/90), respectively. Peptide values were calculated using the labeled peptides that are listed in Table 4.

**TABLE 4:** Results of parallel reaction monitoring compared to those of polymerase chain reaction (PCR) in a validation set.

Enzyme	Peptide	Sensitivity%, CI%, (n MS positive/n PCR positive)	Specificity%, CI%, (n MS negative/n PCR negative)	Sensitivity%, CI%, (n MS positive/n PCR positive) group	Specificity%, CI%, (n MS negative/n PCR negative) group
KPC	AAVPADWAVGDK	100, 91–100 (50/50)	100, 97–100 (138/138)	100, 91–100 (50/50)	100, 97–100 (138/138)
KPC	SQQQAGLLDTPIR	98, 88–100 (49/50)	100, 97–100 (138/138)		
IMP-1	LVVPSHSEVGDASLLK	100, 60–100 (8/8)	100, 97–100 (180/180)	100, 60–100 (8/8)	100, 97–100 (180/180)
IMP-1	VQATNSFSGVNYWLVK	100, 60–100 (8/8)	100, 97–100 (180/180)		
IMP-4	NSFGGVNYWLVK	58, 37–77 (14/24)	100, 97–100 (164/164)	58, 37–77 (14/24)	100, 97–100 (164/164)
IMP	LDEGVYVHTSFEEVNGWGVVPK	92, 73–99 (24/26)	85, 79–90 (138/162)	92, 73–99 (24/26)	85, 79–90 (138/162)
VIM-1	LAEAEGNEIPTHSLEGLSSSGDAVR	100, 20–100 (2/2)	100, 97–100 (186/186)	100, 20–100 (2/2)	100, 97–100 (186/186)
VIM-1	DGDELLLIDTAWGAK	100, 20–100 (2/2)	100, 97–100 (186/186)		
NDM	AFGAAFPK	100, 80–100 (20/20)	100, 97–100 (168/168)	100, 80–100 (20/20)	100, 97–100 (168/168)
OXA-1	NNGLTEAWLESSLK	78, 52–93 (14/18)	100, 97–100 (170/170)	94, 71–100 (17/18)	100, 97–100 (170/170)
OXA-1	IINHNLPVK	78, 52–93 (14/18)	100, 97–100 (170/170)		
CMY-2	ADIANNHPVTQQTLFELGSVSK	27, 12–50 (6/22)	100, 97–100 (166/166)	86, 64–96 (19/22)	100, 97–100 (166/166)
CMY-2	TLQQGIALAQSR	82, 59–94 (18/22)	100, 97–100 (166/166)		
CTX-M-9 group	QLTLGHALGETQR	39, 27–51 (27/70)	100, 96–100 (118/118)	81, 70–89 (57/70)	100, 96–100 (118/118)
CTX-M-9 group	LIAQLGGPGGVTAFAR	81, 70–89 (57/70)	100, 96–100 (118/118)		
CTX-M	TEPTLNTAIPGDPR	98, 93–100 (116/118)	97, 89–100 (68/70)	98, 93–100 (116/118)	97, 89–100 (68/70)
CTX-M-1 group	APLILVTYFTQPQPK	98, 89–100 (57/58)	99, 95–100 (129/130)	98, 90–100 (57/58)	94, 88–97 (122/130)
CTX-M-1 group partial	SDLVNYNPIAEK	76, 83–86 (44/58)	95, 89–98 (123/130)		
TEM	VGYIELDLNSGK	91, 83–96 (89/98)	74, 64–83 (67/90)	91, 83–96 (89/98)	74, 64–83 (67/90)

*PCR, polymerase chain reaction; MS, mass spectrometry.*

## Discussion

For the past few decades, β-lactam antibiotics have been one of the first-choice drugs for the treatment of several serious infectious diseases caused by Gram-negative bacteria (Bush and Bradford, 2016). However, the increasing rate of drug-resistance in bacteria has far exceeded the rate of development of new antibiotics at present. The spread of β-lactamases is mainly attributed to the presence of β-lactamase genes on plasmids, and the unreasonable use of antibiotics. Therefore, a timely detection of the type of β-lactamases will promote appropriate clinical antibiotic choice and inhibit the spread of drug-resistant genes (Iovleva and Doi, 2017). With the development of HRAM Orbitrap MS and supporting quantitative methods, the application of detection approaches based on specific peptides has gradually received attention. Initially, shotgun proteomics was used for β-lactamase detection and the proteins were detected in a single run; however, this depends on the database and results in a poor accuracy (Fleurbaaij et al., 2017). Subsequently, quantitative proteomics methods such as SRM and MRM have been applied to β-lactamases analysis, and detection based on specific peptides has proved to be feasible (Wang et al., 2017). Using LC-MS/MS to detect β-lactamases is a more direct approach than using PCR or disk-diffusion methods, and it is less hindered by multiple problems such as false-positive results. However, for peptide detection, the preparation procedure of peptides must be optimized to reduce the time taken.

In our study, we developed a rapid preparation procedure based on a single colony, thereby omitting the amplification process. Isolating bacteria using agar plates is the first step for isolating organisms from all types of clinical samples. As long as colonies are acquired, the identification process may be initiated. The procedures involving the reduction and alkylation of sulfhydryl groups were also removed as they do not significantly affect the digestion of targeted peptides. Moreover, a simplified Filter-aided sample preparation (FASP) method was used to ensure digestion efficiency. Previous studies have confirmed that microwave treatment for short periods can effectively digest the targeted peptides (Strich et al., 2019). In our study, we tested different digestion conditions for our selected peptides. Results showed that all the targeted peptides could be detected even after microwave treatment for only 5 min, which can greatly reduce the digestion time. For the LC-MS/MS procedure, a 30 min LC method combined with a PRM targeted method was used to identify the peptides. PRM can enable identification of multiple peptides in a high resolution and high mass accuracy mode (Navin, 2015). In contrast with a previous PRM detection method, we used a lower resolution for labeled peptides and higher resolution for targeted peptides to reduce the scanning time and improve the MS/MS quality as the concentration of SIL was high (Stopfer et al., 2021). By optimizing the detection methods, we have obtained a series of peptides with varying properties. Overall, a 30∼60 min preparation procedure, a 30 min LC-MS/MS procedure and a 10 min data processing were determined for the detection of β-lactamases.

For KPC, AAVPADWAVGDK and SQQQAGLLDTPIR were selected as peptides markers (Table 2 and Supplementary Table 2); LALEGLGVNGQ, LTLGSALAAPQR, and APIVLAVYTR were previously analyzed using Agilent 6540 Q-TOF (Wang et al., 2017). NALVPWSPISEK was detected for the first time but was not selected as a marker owing to its low dotp values. Both of the sensitivity and specificity of AAVPADWAVGDK was 100% as it exists in all the genotypes of *bla*_*KPC*_, indicating the possibility of becoming a peptide marker for KPC. But for SQQQAGLLDTPIR, the sensitivity was lower as the absence of *bla_*KPC–*13_*, *bla_*KPC–*45_*, and*bla_*KPC–*59_*. This study shows the first successful detection of IMP *via* LC-MS/MS. The results proved that LVVPSHSEVGDASLLK, VQATNSFSGVNYWLVK, and NSFGGVNYWLVK could effectively be used to identify IMP and distinguish the subtypes of *bla_*IMP–*1_* and *bla_*IMP–*4_*. LDEGVYVHTSFEEVNGWGVVPK showed lower specificity for IMP detection. And for NSFGGVNYWLVK, it is inexplicable that the discrepant results were verified to be *bla_*IMP–*4_* positive, while the Skyline map fragments irons of the discrepant results was different from the positive ones. This is the first time this phenomenon has been observed, and it may be related to the fragmentation of the peptide. Notably, LVVPSHSEAGDASLLK (IMP-4), whose A was replaced by V in IMP-1 (Δ14Da), was also detected and could be separated *via* high-resolution MS. LAEAEGNEIPTHSLEGLSSSGDAVR and DGDELLLIDTAWGAK were used to detect VIM-1, and both of them showed a better subtype coverage. However, as the lack of *bla_*VIM–*1_* strains collected, only 2 *bla_*VIM–*1_* positive strains were identified in 188 strains. With the continuous collection of *bla_*VIM–*1_* positive strains, the effectiveness of detection by peptide markers can be better verified in the future. AFGAAFPK was selected as the marker as it exists in all NDM subtypes currently listed by the NCBI, and was also detected *via* the MRM targeted method (Wang et al., 2019). *bla*_*OXA*_ is widely distributed in *Enterobacteriaceae*. Regarding the *bla_*OXA–*1_* family, four peptides were detected, and NNGLTEAWLESSLK and IINHNLPVK, which were specific for the *bla_*OXA–*1_* family were used for screening. However, according to the reproducibility results, the LC-MS/MS signal of IINHNLPVK was not stable in the detection process. *bla*_*CMY*_ is an AmpC type ESBL gene. ADIANNHPVTQQTLFELGSVSK and TLQQGIALAQSR showed 27 and 82% sensitivity for the strains used in this study. However, for TEM (including TEM-1, TEM-2, and variants listed on the NCBI ANTIMICROBIAL RESISTANCE GENE database), LLTGELLTLASR, SALPAGWFIADK, IHYSQNDLVEYSPVTEK, QIAEIGASLIK, VDAGQEQLGR, VDAGQEQLGRR, and VGYIELDLNSGK were identified in DDA experiments (Supplementary Table 2). QIAEIGASLIK and VGYIELDLNSGK were choose as peptides markers, and QIAEIGASLIK was removed for its poor spectrum. For the 188 strains detected, VGYIELDLNSGK showed 91% sensitivity with lower specificity as 74% (67/90). Overall, this is the first study to use a PRM-based LC-MS/MS system to detect OXA, CMY, and TEM. *bla*_CTX–M_ ESBL is a large group of ESBLs with an increasing number of subtypes. According to genetic structure, *bla*_CTX–M_ enzymes are divided into four groups (CTX-M-1, CTX-M-2, CTX-M-9, and CTX-M-25) (Canton et al., 2012). Among them, the CTX-M-15 (CTX-M-1 group) and CTX-M-14 (CTX-M-9 group) are by far the most prevalent enzymes. In our study, only APLILVTYFTQPQPK showed 98% sensitivity for the CTX-M-1 group. QLTLGHALGETQR and LIAQLGGPGGVTAFAR only existed in certain strains of the CTX-M-9 group, whereas SDLVNYNPIAEK showed 76% sensitivity for the CTX-M-1 group. Additionally, TEPTLNTAIPGDPR could identify 98% of the strains containing CTX-M-1 or CTX-M-9 groups. The reason for the relatively lower sensitivity may be that the peptides are not the representative peptides for the entire genotype group, but only for the partial group due to the complexity of the variants. It may not be possible to use single peptide to achieve 100% sensitivity. As the overall detection sensitivity for each drug resistant enzyme is more important than the detection sensitivity of individual peptide, to overcome this complexity of huge number of variants, sensitivity and specificity based on peptides groups were calculated as shown in Table 4. The sensitivity of KPC, OXA, CMY-2, CTX-M-9 group, and CTX-M-1 peptides groups raised to 100, 94, 76, 81, and 98%. In addition, much more peptide markers need to be effectively detected and added for the group detection in the future study. In addition, several variants were observed for the peptides of *bla*_CTX–M_, such as LGVALIDTADNTQVLYR, LGVALINTADNTQTLYR, and LGVALINTADNSQILYR, which are similar in terms of m/z and RT, representing great challenges to detection *via* MS. Therefore, to improve the sensitivity of detection *via* LC-MS/MS, an overall analysis is suggested using all the peptides in one method. Further studies are required to identify specific peptides for one group or the entire *bla*_CTX–M_ group.

The dotp is a measure of similarity between the fragment ratio of the endogenous and library peptide. The rdotp is a measure of similarity between the fragment ratio of the endogenous and SIL peptide. The criterium for judging positivity by a previous study was rdotp value > 0.95 (Foudraine et al., 2019). When we re-analyzed the results of the targeted proteomics experiment, we found that unified screening rules might not have been suitable for all peptides. For many peptides such as LDEGVYVHTSFEEVNGWGVVPK (IMP), DGDELLLIDTAWGAK (VIM-1), and TEPTLNTAIPGDPR (CTX-M-1 group partial), a rdotp value > 0.95 is not enough to screen for positive strains; therefore, library dotp values > 0.8 are required. For QLTLGHALGETQR (CTX-M-9), 100% (70/70) strains were positive for library dotp > 0.8 compared to PCR results; however, only 39% (27/70) strain was positive based on values of rdotp > 0.95 and library dotp > 0.8. For LIAQLGGPGGVTAFAR (CTX-M-9), 100% (70/70) strains were positive for library dotp > 0.8 compared to PCR results; however, only 81% (57/70) strains were positive based on values of rdotp > 0.95 and library dotp > 0.8. This phenomenon is partly due to the existence of variants.

Overall, directly identifying peptides *via* LC-MS/MS provides a new approach to detect β-lactamases. Our results showed the great potential of the rapid extraction and detection method in the detection of β-lactamases. Started by picking the colony, peptides were obtained by rapid ultrasonic lysis and rapid digestion (even in a microwave oven for 5 min), and then results were acquired by rapid LC-MS/MS analysis (30 min) and data processing. The application of targeted proteomics using single bacterial colonies and even blood samples in the future, will enable early clinical diagnostics and early treatment. However, there are several problems that need to be addressed. For example, at present, drug-resistant enzymes are named and classified based on gene sequence; however, the protein sequence is more decisive in determining function. Additionally, there is a lack of protein subtypes and classification based on amino acid sequences. Therefore, it is difficult to identify specific peptides for one group or one subtype. Variants are ubiquitous in β-lactamase enzymes such as *bla*_CTX–M_. The separation of variants with a similar m/z and RT through regular LC-MS/MS remains a large challenge.

In conclusion, the rapid and accurate identification of β-lactamases is of great significance to clinical diagnostics and treatment. This study describes a rapid extraction and detection workflow for widespread β-lactamases, including KPC, IMP, VIM, NDM, OXA, CMY, CTX-M, and TEM using single colonies of *Enterobacteriaceae* strains. PRM targeted proteomics was proven to be a promising approach for the detection of drug-resistant enzymes.

## Data Availability Statement

The datasets presented in this study can be found in online repositories. The names of the repository/repositories and accession number(s) can be found in the article/Supplementary Material.

## Author Contributions

XFY and YL: conceptualization and funding acquisition. YL, XH, XW, and JP: methodology. YL and GL: software. YL: validation, formal analysis, writing—original draft preparation, and supervision. XH: resources. XYY, XFY, and CL: writing—review and editing. GL: visualization. XFY: project administration. All authors have read and agreed to the published version of the manuscript.

## Conflict of Interest

The authors declare that the research was conducted in the absence of any commercial or financial relationships that could be construed as a potential conflict of interest.

## Publisher’s Note

All claims expressed in this article are solely those of the authors and do not necessarily represent those of their affiliated organizations, or those of the publisher, the editors and the reviewers. Any product that may be evaluated in this article, or claim that may be made by its manufacturer, is not guaranteed or endorsed by the publisher.

## Footnotes

1
https://www.ncbi.nlm.nih.gov/pathogens/refgene/

2
https://blast.ncbi.nlm.nih.gov/Blast.cgi?PROGRAM=blastp&PAGE_TYPE=BlastSearch&LINK_LOC=blasthome

3
https://www.ncbi.nlm.nih.gov/protein/

4
https://web.expasy.org/peptide_cutter/
