# Supplementary material for: Parallel Reaction Monitoring Mass Spectrometry for Rapid and Accurate Identification of β-Lactamases Produced by Enterobacteriaceae
Source: Front Microbiol. 2022 Jun 20;13:784628. doi: 10.3389/fmicb.2022.784628 (PMC9251374; doi:10.3389/fmicb.2022.784628)
Supplement: Supplementary file 1 [file Table_1.DOCX]

Supplementary Material

**Supplementary Table 1.** Primers used for the PCR assays**^(1)^。**

| \| **PCR name** \| \| --- \| | **β-Lactamase(s) targeted** | **Sequence (5’–3’)** | \|  \| **Amplicon size (bp)** \| \| --- \| --- \| |
| --- | --- | --- | --- | --- | --- | --- |
| Multiplex I | KPC-1 to KPC-5 | CATTCAAGGGCTTTCTTGCTGC | 538 |
|  |  | ACGACGGCATAGTCATTTGC | 538 |
|  | IMP variants except IMP-9, IMP-16,  IMP-18, IMP-22 and IMP-25 | TTGACACTCCATTTACDG | 139 |
|  |  | GATYGAGAATTAAGCCACYCT | 139 |
|  | VIM variants including VIM-1 and VIM-2 | GATGGTGTTTGGTCGCATA | 390 |
|  |  | CGAATGCGCAGCACCAG | 390 |
| \| Multiplex II \| \| --- \| | TEM variants including TEM-1 and TEM-2 | CATTTCCGTGTCGCCCTTATTC | 800 |
|  |  | CGTTCATCCATAGTTGCCTGAC | 800 |
|  | LAT-1 to LAT-3, BIL-1, CMY-2 to CMY-7,  CMY-12 to CMY-18 and CMY-21 to  CMY-23 | CGAAGAGGCAATGACCAGAC | 538 |
|  |  | ACGGACAGGGTTAGGATAGY | 538 |
|  | NDM-1,9,12,13,14,29 | ATTCGCCCCATATTTTTGCTA | 1013 |
|  |  | GATCCTTCCAACTCGTCGCA | 1013 |
| \| Multiplex III \| \| --- \| | variants of CTX-M group 1 including  CTX-M-1, CTX-M-3 and CTX-M-15 | TTAGGAARTGTGCCGCTGYA | 688 |
|  |  | CGATATCGTTGGTGGTRCCAT | 688 |
|  | variants of CTX-M group 9 including  CTX-M-9 and CTX-M-14 | TCAAGCCTGCCGATCTGGT | 561 |
|  |  | TGATTCTCGCCGCTGAAG | 561 |
| Multiplex IV | OXA-1, OXA-4 and OXA-30 | GGCACCAGATTCAACTTTCAAG | 564 |
|  |  | GACCCCAAGTTTCCTGTAAGTG | 564 |
| *bla_IMP-1_* | IMP-1 | CAAGCGCGTTACGCCGTGGGT | 939 |
|  |  | TTGAAGTTGCGCGTTGTGGA | 939 |
| *bla_IMP-4_* | IMP-4 | ATGAGCAAGTTATCTGTATT | 741 |
|  |  | TTAGTTGCTTAGTTTTGATGG |  |

(1). Dallenne C, Costa AD, Decré D, Favier C. 2010. Development of a set of multiplex PCR assays for the detection of genes encoding important beta-lactamases in Enterobacteriaceae. Journal of Antimicrobial Chemotherapy 65:490.
