# Supplementary material for: Parallel Reaction Monitoring Mass Spectrometry for Rapid and Accurate Identification of β-Lactamases Produced by Enterobacteriaceae
Source: Front Microbiol. 2022 Jun 20;13:784628. doi: 10.3389/fmicb.2022.784628 (PMC9251374; doi:10.3389/fmicb.2022.784628)
Supplement: Supplementary file 2 [file Table_2.DOCX]

Supplementary Material

**Supplementary Table 2.** Candidate peptides for β-lactamases.

| Enzyme | Genetype^a^ | Peptide |
| --- | --- | --- |
| KPC | All except for 52,55 | APIVLAVYTR |
| KPC | All | GFLAAAVLAR |
| KPC | All except for 4,5,10,11,15,21,22,27 | NALVPWSPISEK |
| KPC | All except for19,56,38 | LALEGLGVNGQ |
| KPC | All except for 16 | LTLGSALAAPQR |
| IMP | 1,2,3,4,5,6,7, etc. | SIPTYASELTNELLK |
| IMP | 1,2,3,4,5,6,7, etc. | SIPTYASELTNELLKK |
| IMP-1 | 1,10,6,25,30,40,42,52,55,60,66,70,76~80,88 | GSISSHFHSDSTGGIEWLNSR |
| IMP-1 | 1,10,6,34,3,30,40,42,52,55,60,61,66,70, 76~80,88 | LGDANIEAWPK |
| IMP-4 | 59,4,26,89 | LVVPSHSEAGDASLLK |
| IMP | 1,3,4,5,6,7,10 etc. | LVTWFVER |
| VIM | All except for 7,5,25,38,49,8,9,61,69,66,71 | AAGVATYASPSTR |
| VIM | All except for 7,5,25,38,49,8,9,61,69,66,71 | AAGVATYASPSTRR |
| VIM | All except for 71 | QIGLPVTR |
| NDM | 6,12,15,19,27, 26 | SLGNLGDADTEHYAASAR |
| OXA-1  family | 1,4,31,47,224,320,392,534,675(oxa-1family) | ISPEEQIQFLR |
| OXA-1  family | 1,4,31,47,224,320,392,534,675(oxa-1family) | TGAGFTANR |
| CMY-2 family | All except for BIL-1,LAT-1,145,60,55,36 | TFNGVLGGDAIAR |
| CMY-2 family | All except for 37 | VALAAIPAVEVNPPAPAVK |
| CMY-2 family | All except for 71,80,102,96,104,155 | TEQQIADIVNR |
| CMY-2 family | All except for 144,40,45,49 | TGSTGGFGSYVAFVPEK |
| CTX-M | 9,13,14,17, 19,21,24, etc. | TGSGDYGTTNDIAVIWPQGR |
| CTX-M | 9,13,14,17, 19,21, etc. | APLVLVTYFTQPQQNAESRR |
| CTX-M | 9,13,14,17, 19,21, etc. | APLVLVTYFTQPQQNAESR |
| CTX-M | 13,14, 17,19, 21,etc. | AGLPTSWTVGDK |
| CTX-M-81 | 81 | QLLNQPVEIQPADLVNYNPIAEK |
| CTX-M | 65,24,148,122,130,196,99 | APLVLVTYFTQPQQNAER |
| CTX-M | 1, 10,15,23,etc. | SESEPNLLNQR |
| CTX-M | 3,10,12,15,23,etc. | LIAHVGGPASVTAFAR |
| CTX-M | 15,28,29,etc. | TGSGGYGTTNDIAVIWPK |
| CTX-M | 1,10,12,15,23,etc. | GNTTGAASIQAGLPASWVVGDK |
| CTX-M | 9,14,16,17, 19,21,24, 81,etc. | LGVALIDTADNTQVLYR |
| CTX-M | 25,26,39,41,10,94,185,160, 89,91,217,78,205,152 | LGVALINTADNTQTLYR |
| CTX-M | 1,2,3,5,10,12,15,23,etc. | LGVALINTADNSQILYR |
| TEM | All except for 70,88,108,194 | LLTGELLTLASR |
| TEM | All except for 60,83,147,167,176,183,226 | SALPAGWFIADK |
| TEM | 1,2,10,etc. | IHYSQNDLVEYSPVTEK |
| TEM | All except for 101,151,227,164,104 | QIAEIGASLIK |
| TEM | All except for 116,171,229,205,57,157,233,162 | VDAGQEQLGR |
| TEM | All except for 116,171,229,205,57,157,233,162 | VDAGQEQLGRR |

a All the genetype matching results were depends on the NCBI ANTIMICROBIAL RESISTANCE GENE database. With the increasing data quantity, matching results will changed.
