# Supplementary material for: Parallel Reaction Monitoring Mass Spectrometry for Rapid and Accurate Identification of β-Lactamases Produced by Enterobacteriaceae
Source: Front Microbiol. 2022 Jun 20;13:784628. doi: 10.3389/fmicb.2022.784628 (PMC9251374; doi:10.3389/fmicb.2022.784628)
Supplement: Supplementary file 4 [file Data_Sheet_1.docx]

Supplementary Material





**Supplementary Figure 1.** Peptides detected under different digestion methods.
