# Supplementary figures and images for: Parallel Reaction Monitoring Mass Spectrometry for Rapid and Accurate Identification of β-Lactamases Produced by Enterobacteriaceae
Source: Front Microbiol. 2022 Jun 20;13:784628. doi: 10.3389/fmicb.2022.784628 (PMC9251374; doi:10.3389/fmicb.2022.784628)

Supplementary Material
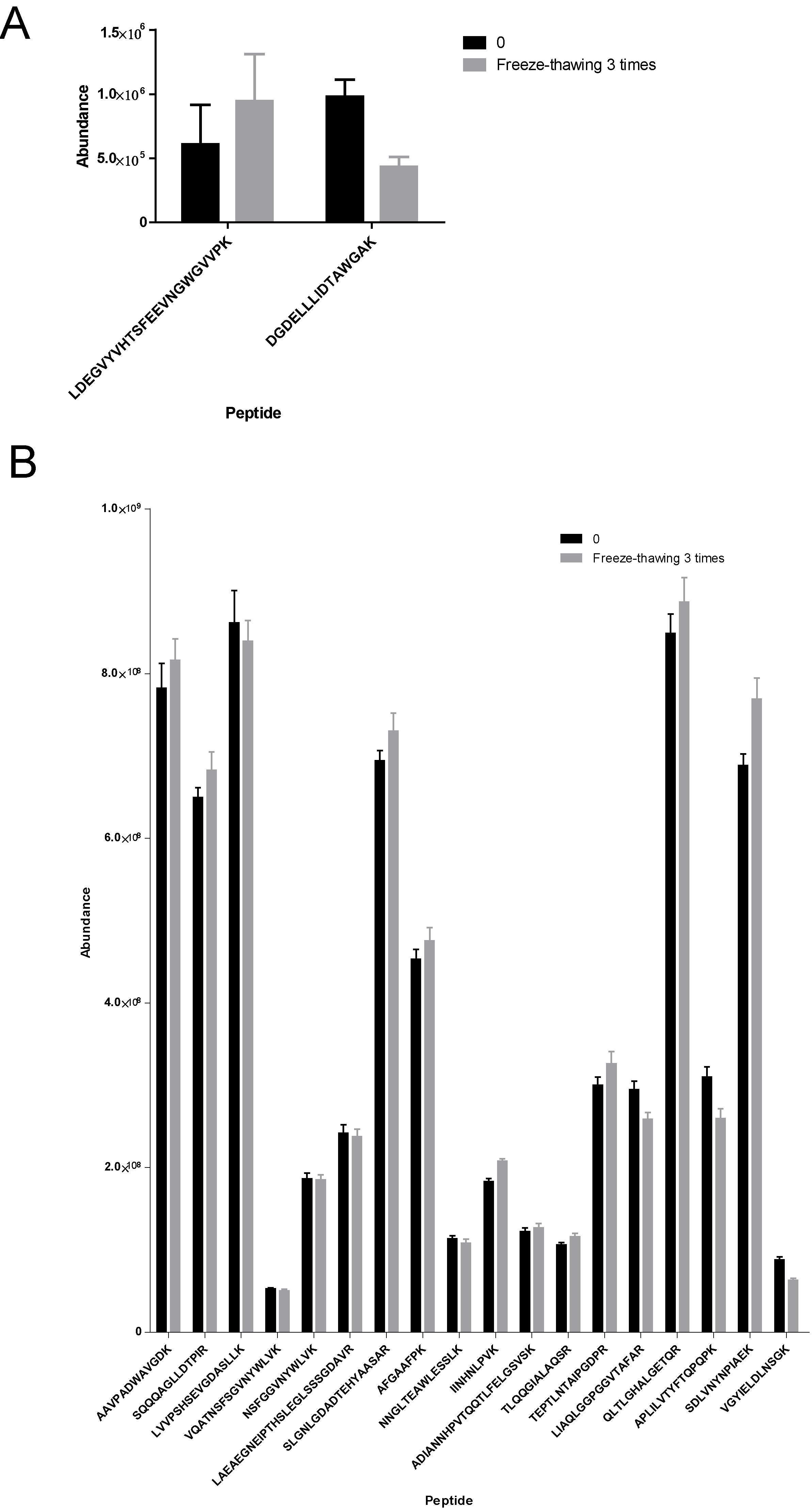


**Supplementary Figure 2.** Freeze-thaw stability of selected peptides.

Supplement: Supplementary file 5 [file Data_Sheet_2.docx]
