# Supplementary material for: Parallel Reaction Monitoring Mass Spectrometry for Rapid and Accurate Identification of β-Lactamases Produced by Enterobacteriaceae
Source: Front Microbiol. 2022 Jun 20;13:784628. doi: 10.3389/fmicb.2022.784628 (PMC9251374; doi:10.3389/fmicb.2022.784628)
Supplement: Supplementary file 6 [file Data_Sheet_3.docx]

Supplementary Material


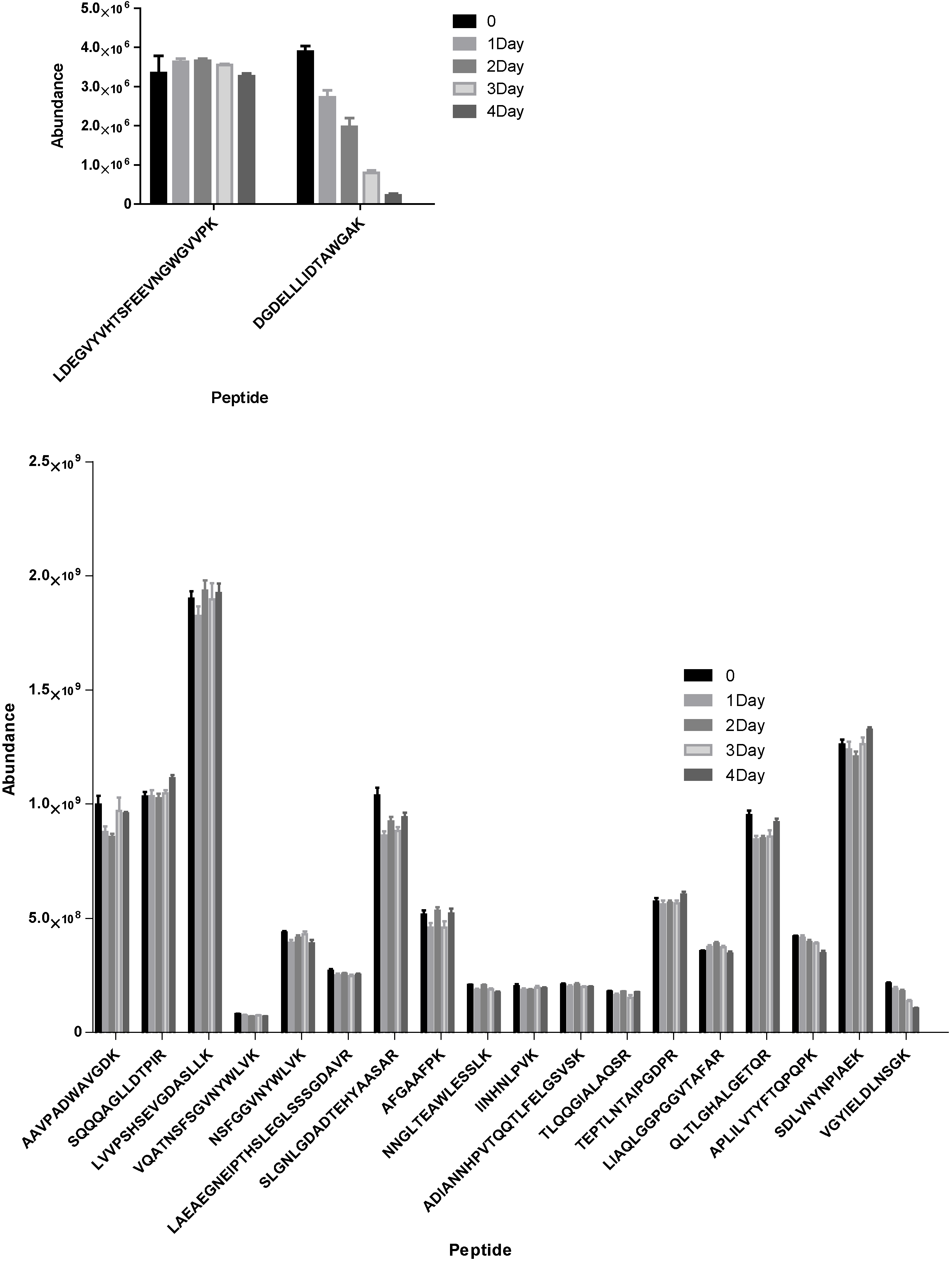


**Supplementary Figure 3.** Stability of selected peptides in sample holder.
